# Comparative electronic structures of nitrogenase FeMoco and FeVco†
†Electronic supplementary information (ESI) available: Additional figures and tables, computational data and information. See DOI: 10.1039/c7dt00128b



**DOI:** 10.1039/c7dt00128b

**Published:** 2017-02-02

**Authors:** Julian A. Rees, Ragnar Bjornsson, Joanna K. Kowalska, Frederico A. Lima, Julia Schlesier, Daniel Sippel, Thomas Weyhermüller, Oliver Einsle, Julie A. Kovacs, Serena DeBeer

**Affiliations:** a Max Planck Institute for Chemical Energy Conversion , Stiftstr. 34-36 , 45470 Mülheim an der Ruhr , Germany . Email: serena.debeer@cec.mpg.de ; Tel: +49 208 306 3605; b Department of Chemistry , University of Washington , Box 351700 , Seattle , WA 98195-1700 , USA . Email: kovacs@chem.washington.edu; c Science Institute , University of Iceland , Dunhagi 3 , 107 Reykjavik , Iceland; d Centro Nacional de Pesquisa em Energia e Materiais Brazilian Synchrotron Light Laboratory - LNLS Rua Giuseppe Máximo Scolfaro , 10.000 13083-970 Campinas SP , Brazil; e Institute for Biochemistry and BIOSS Centre for Biological Signalling Studies , Albert Ludwigs University Freiburg , Germany . Email: einsle@bio.chemie.uni-freiburg.de; f Department of Chemistry and Chemical Biology , Cornell University , Ithaca , NY 14853 , USA

## Abstract

An investigation of the active site cofactors of the molybdenum and vanadium nitrogenases (FeMoco and FeVco) was performed using high-resolution X-ray spectroscopy. Synthetic heterometallic iron–sulfur cluster models and density functional theory calculations complement the study of the MoFe and VFe holoproteins using both non-resonant and resonant X-ray emission spectroscopy. Spectroscopic data show the presence of direct iron–heterometal bonds, which are found to be weaker in FeVco. Furthermore, the interstitial carbide is found to perturb the electronic structures of the cofactors through highly covalent Fe–C bonding. The implications of these conclusions are discussed in light of the differential reactivity of the molybdenum and vanadium nitrogenases towards various substrates. Possible functional roles for both the heterometal and the interstitial carbide are detailed.

## Abstract 2

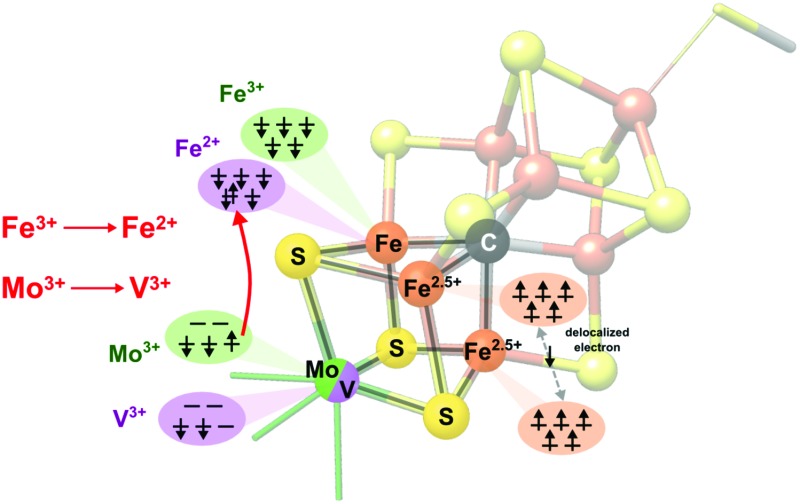
High-resolution X-ray spectroscopy provides insights into the electronic structural differences between the nitrogenase FeMoco and FeVco clusters.

## Introduction

**Fig. 1:**
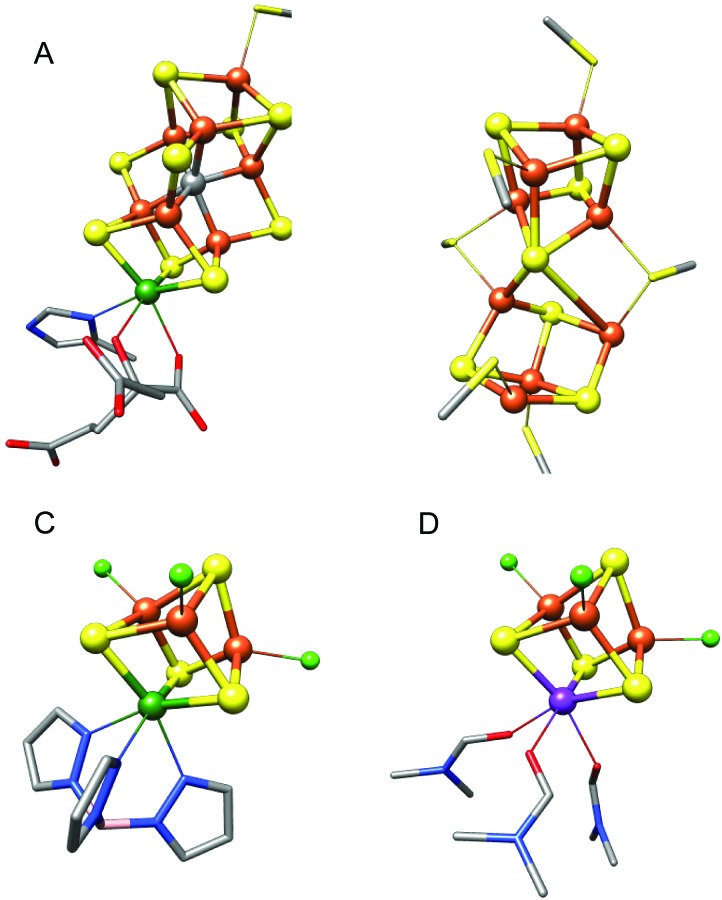
Structural representations of FeMoco (A) and the P-cluster (B) from *Azotobacter vinelandii* nitrogenase, adapted from PDB ; 3U7Q, and the synthetic cubane molybdenum and vanadium iron–sulfur cluster models (Et_4_N)[(Tp)MoFe_3_S_4_Cl_3_]^18^ (C) and (Me_4_N)[VFe_3_S_4_Cl_3_(DMF)_3_]^19^ (D) used in this study. Tp = tris(pyrazolyl)borate, DMF = dimethylformamide. Color scheme for atoms: Fe = orange, S = yellow, C = gray, Mo = green, V = purple, N = blue, O = red, Cl = light green, B = pink. Inorganic sulfides are shown as spheres, cysteinate residues are shown as sticks, and hydrogen atoms and counterions have been omitted for clarity.

The nitrogenase enzymes catalyze the reduction of atmospheric dinitrogen. Of the three classes of nitrogenases, delineated by the elemental composition of their active site cofactors, the molybdenum-dependent nitrogenases are the most active under ambient conditions.^1^ They are also the most well-studied; a combination of crystallographic^2^ and spectroscopic^2,3^ studies have provided the complete atomic structure of the MoFe_7_S_9_C iron–molybdenum cofactor, (FeMoco) as shown in Fig. 1A. The FeMoco is located in the MoFe protein, which also contains an Fe_8_S_7_ cluster termed the P-cluster (Fig. 1B). While both of these unique iron–sulfur clusters are essential for the function of nitrogenase, the presence of 15 total iron atoms in the clusters complicates the interpretation of spectra obtained using element-specific techniques such as ^57^Fe Mössbauer and X-ray spectroscopies. Thus, the exact electronic structure of FeMoco remains an area of active investigation.^4–8^

In contrast, the vanadium-dependent nitrogenase is a less efficient N_2_ reduction catalyst at ambient temperatures, requiring more ATP and additional reducing equivalents (Table S1†). However, its activity is comparably unaffected by decreased temperatures.^9^ Furthermore, it has been shown that the vanadium nitrogenase exhibits a unique ability to promote the reduction of CO to short-chain hydrocarbons (Table S1†).^10^ This reductive C–C bond coupling is akin to Fischer–Tropsch chemistry, but utilizes protons and electrons in place of H_2_. The relative inability of the molybdenum nitrogenase to perform the same chemistry (CO is a reversible inhibitor of molybdenum nitrogenase^11^) poses significant questions regarding the structural and/or electronic differences underlying the disparate reactivity of these two isozymes.

We have recently provided the first direct evidence for an interstitial carbide in the iron–vanadium cofactor (FeVco),^12^ establishing the structural homology of FeMoco and FeVco (the so-called M-clusters, where M = Mo or V). A subsequent, independent study confirmed our findings and also reported the binding of CO to the resting state of FeVco.^13^ The latter result is of significant interest because CO coordination to FeMoco only occurs under turnover conditions. This suggests that despite the similar geometric structures of the M-clusters, they have sufficiently different electronic structures so as to impact their ability to bind substrate. It is possible that these changes in the electronic structure also engender the differences in reactivity towards native and non-native substrates detailed above.

To address these questions, the present work examines the comparative electronic structures of the M-clusters in the intact MoFe and VFe proteins. A combination of high-energy resolution fluorescence detected (HERFD) X-ray absorption spectroscopy (XAS) at the Kα (2p → 1s) and Kβ (3p → 1s) emission lines, as well as non-resonant Kβ X-ray emission spectroscopy (XES) is utilized in order to obtain detailed insight into the electronic structural differences in the protein active sites. By comparing the present data to previously published data^3,14^ on both a P-cluster-only variant of nitrogenase (Δ*nifB*), as well as isolated FeMoco, we are able to ascertain which spectral features correspond to the M-cluster *vs.* the P-cluster. Further insight into the role of the heterometal in tuning the electronic structure of the M-clusters is obtained by parallel HERFD XAS and non-resonant XES studies on synthetic, single-cubane MoFe_3_S_4_ and VFe_3_S_4_ clusters (Fig. 1C and D). Through correlation of the experimental XAS and XES studies to density functional theory (DFT) calculations, a quantitative picture of the electronic structure differences between FeMoco and FeVco emerges. The results provide evidence for changes in M-cluster oxidation states, as well as differences in intra-cluster bonding and covalency. The role of these electronic structural differences in tuning substrate binding affinities is discussed.

## Experimental

### Sample preparation

The MoFe^2^ and VFe^15^ proteins were expressed and purified as previously described, and samples for X-ray spectroscopic measurements were also prepared following published protocols.^12^ FeMoco was extracted from MoFe protein by treatment with *N*-methylformamide (NMF) and dithiothreitol (DTT), following the protocol of Shah and Brill.^16,17^ The extraction of FeMoco was performed using 1 mL of NMF for 25 mg of protein, and used under strict exclusion of dioxygen, without further concentration. The final concentration of cofactor is estimated to be ∼0.2 mM. An aliquot of the NMF and DTT solution of extracted cofactor was placed in a Delrin cell with a 38 μm Kapton tape window, and immediately frozen in liquid N_2_.

The model compounds (Et_4_N)[(Tp)MoFe_3_S_4_Cl_3_]^18^ and (Me_4_N)[VFe_3_S_4_Cl_3_(DMF)]^19^ were synthesized as previously reported, and handled under inert atmosphere. Samples were prepared by grinding solid compound into a fine powder in a mortar and pestle. The powders were then pressed into 1 mm thick Al spacers and sealed with 38 μm Kapton tape. For non-resonant XES measurements, the solid samples were measured without dilution, and for HERFD XAS measurements samples were diluted ∼1 : 10 by mass with BN to minimize self-absorption.

### Data collection

X-ray spectroscopic measurements were conducted at beamline ID-26 at the European Synchrotron Radiation Facility (ESRF), France, and at beamline C-1 at the Cornell High Energy Synchrotron Source (CHESS), NY, USA. For all experiments, the flight path of the emitted X-rays was filled with He gas to minimize signal attenuation. Samples were maintained at cryogenic temperatures, using a continuous flow liquid He cryostat at 10 K at ESRF and a He displex cryostat at 40 K at CHESS. For all experiments, the monochromatic incident energy was calibrated to the first inflection point of an iron foil (7111.2 eV), and the XES spectrometers were calibrated using the Kβ emission features of Fe_2_O_3_ (Kβ′ = 7045.2 eV, Kβ_1,3_ = 7060.6 eV, Kβ′′ = 7092.0 eV, Kβ_2,5_ = 7107.2 eV).

Non-resonant Fe Kβ XES measurements were performed at both ESRF and CHESS. At ESRF, the incident energy was set to 7800 eV, selected using a Si(111) double crystal monochromator, with a storage ring electron current of approximately 200 mA and energy of 6 GeV. The photon flux at the sample was approximately 10^13^ photons per s, with a beam spot on the sample of 0.1 mm × 1 mm (vert. × hor.). Kβ fluorescence was analyzed using a Johann-type spectrometer, employing five spherically bent Ge(620) crystals and a dead-time corrected Ketek Si drift diode detector, configured in a Rowland geometry as described previously.^20^ At CHESS, the incident energy was set to approximately 9000 eV, selected using a pair of Mo/B_4_C multilayers for approximately 1% bandpass, with a storage ring electron current of 85 mA operating in 90 minute decay mode. Photon flux at the sample was approximately 2 × 10^12^ photons per s, with a beam spot size of 1 mm × 2 mm (vert. × hor.). Fe Kβ fluorescence was analyzed using DAVES,^21^ the dual-array valence emission spectrometer, using five spherically-bent Ge(620) crystals similarly arranged in a Johann-type configuration. Analyzed emission was captured on a Pilatus 100 K detector (Dectris) in a Rowland geometry, and a digital region of interest was selected to tightly enclose the reflections from all 5 analyzer crystals.

Resonant Fe Kβ XES measurements were also performed at ESRF and CHESS. At CHESS, the multilayers were replaced by a Si(111) monochromator, and photon flux decreased to approximately 10^10^ photons per s. At both beamlines, HERFD XAS spectra were obtained by scanning the incident energy while detecting narrow bandwidth fluorescence at either the maximum of the Kβ_1,3_ or Kβ′ emission features (Table S2†). Initially, spectra were recorded from 7080 eV to 7600 eV to include the extended X-ray absorption fine structure (EXAFS) region for normalization. Repeated spectra were then collected from 7080 eV to 7200 eV to improve the data quality (signal-to-noise ratio, S/N) in the pre-edge and edge regions.

Resonant Fe Kα XES measurements were performed at beamline ID-26 at ESRF. For these experiments, the incident energy was selected using a Si(311) double crystal monochromator, with a storage ring electron current of approximately 90 mA and energy of 6 GeV in 16-bunch operating mode. Photon flux at the sample was approximately 5 × 10^11^ photons per s, with a beam spot size of 0.1 mm × 0.7 mm (vert. × hor). Kα fluorescence was analyzed using the same Johann-type spectrometer, employing four Ge(440) crystals and a Ketek detector. HERFD XAS spectra were obtained by scanning the incident energy while detecting narrow bandwidth fluorescence at the maximum of the Kα_1_ emission line (6404 eV). For normalization, spectra including the EXAFS region were collected from 7000 eV to 8000 eV. Repeated spectra were then collected from 7105 eV to 7180 eV to improve the S/N in the pre-edge and edge regions.

Radiation damage assessments were conducted at the beginning of data collection for each sample. To determine the acceptable dwell time per sample spot, rapid Fe Kβ (resp. Kα) HERFD XAS spectra were recorded on the same location, scanning over the edge region. At ESRF, the XAS spectra were found to be superimposable up to (and in some cases well beyond) 25 (resp. 50) seconds of beam exposure, which was chosen as the maximum irradiation time. The cubane model complexes were found to be more susceptible to radiation damage than the proteins. Accordingly, while data with poor S/N were obtained on the synthetic cubane cluster models at ESRF, the Kβ XES and Kβ HERFD XAS spectra from CHESS provide improved S/N without evidence of radiation damage. The smaller photon flux density (flux/beam spot area) due to a larger beam spot size is expected to decelerate damage.^22^

### Data analysis

For all experiments, individual scans of the same sample were normalized to the incident photon flux and averaged using PyMCA.^23^ The averaged spectra were further processed using MATLAB. The energy axes of the non-resonant Kβ XES spectra for the present data, as well as for previously reported data, *infra*,^3,14^ were compared using Fe_2_O_3_ as a common energy reference (as noted in the data collection). Following energy calibration (Fig. S1†), the integrated intensities of the Fe Kβ XES spectra were normalized to 100.

The averaged resonant Fe Kβ XES data were plotted as narrow bandwidth fluorescence yield (detector counts) at the desired emission feature, as a function of incident energy, to obtain HERFD XAS spectra. Spectra that included the EXAFS region were plotted and the intensity was scaled to achieve superimposable post-edge data for all spectra. The high-energy side of all the spectra was normalized to unity (Fig. S2†). The corresponding spectrum of each sample which encompassed just the edge region, with superior S/N, was then scaled such that it overlayed the pre-edge and edge of the normalized EXAFS traces. The energy positions of the pre-edge and edge features were determined from plots of the first derivatives of all spectra. These were obtained by simultaneous smoothing and differentiation, using the Savitzky–Golay filter as implemented in EasySpin 5.0.2.^24^ Plots of the first derivatives of the Kα-detected HERFD XAS and Kβ XES spectra are provided in Fig. S3 and S4,† respectively.

### DFT calculations

All calculations were performed using the ORCA program packages developed by Neese and coworkers.^25^ 225 atom cluster models of the FeMoco and FeVco active sites, based on the X-ray structure of the MoFe protein,^2^ were TPSSh-optimized as previously described.^4,12^ Charges on the metal clusters were –1 for FeMoco ([MoFe_7_S_9_C]^1–^) and –2 for FeVco ([VFe_7_S_9_C]^2–^), to maintain a valence isoelectronic configuration and to be consistent with the experimentally-determined spin state of *S* = 3/2 for both cofactors.^11^ Analogous *M*_s_ = 3/2 broken-symmetry solutions were found for both FeMoco and FeVco, as previously discussed in ref. 4, 7 and 12. Identical procedures were utilized for the synthetic cubane cluster models, with geometry optimizations initiated from the crystallographic coordinates. TD-DFT Fe XAS calculations used the BP86 functional^26,27^ and the DKH relativistic approximation,^28–30^ with DKH-recontracted def2-TZVP triple-zeta basis sets^31,32^ and the COSMO dielectric model (*ε* = 4).^33^ TD-DFT calculations for the XAS spectra of Fe pre-edges were performed using previously reported protocols.^34^ The donor orbitals for XAS calculations were chosen as the Fe 1s and all virtual orbitals were selected as possible acceptor orbitals. Up to 300 roots were calculated. Individual Fe 1s excitations from all component irons were averaged to obtain the cluster spectra.

## Results

### Electronic structure calculations

**Fig. 2:**
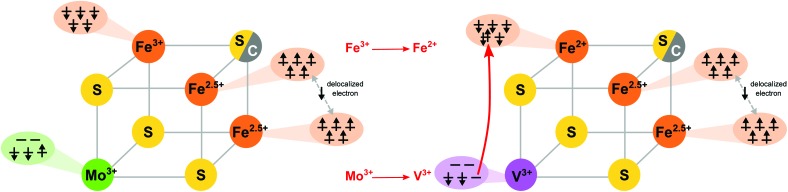
Spin coupling diagram for Mo (left) and V (right) protein cofactors and synthetic MoFe_3_S_4_ and VFe_3_S_4_ cubane models. In all four cases, DFT calculations predict a trivalent heterometal. The spin-up electron on the non-Hund Mo(iii) ion is found to relocate to the antiferromagnetically-coupled iron in the vanadium clusters, resulting in a reduction from Fe^3+^ to Fe^2+^.

Before presenting the experimental results, it is instructive to first examine the results of the electronic structure calculations. Broken-symmetry DFT calculations were utilized in order to derive qualitative spin coupling diagrams for the cofactors and cubane cluster models. Fig. 2 shows a comparison of the simplified coupling schemes for FeMoco/the MoFe_3_S_4_ cubane cluster (left) and for FeVco/the VFe_3_S_4_ cubane cluster (right). As reported previously, both FeMoco and the synthetic MoFe_3_S_4_ cubane cluster have identical coupling schemes, with the Mo^3+^ ion in both the cofactor and the model having an unusual non-Hund configuration.^4^ The remaining three iron ions (focusing on the lower half of the cofactor as depicted in Fig. 1) are arranged in a spin-up (by convention) ferromagnetically-coupled Fe^2.5+^ pair, sharing a delocalized spin-down electron, and an antiferromagnetically-coupled Fe^3+^.

The spin coupling arrangement in FeVco and the VFe_3_S_4_ cubane cluster are also similar to each other, and clearly distinct from the Mo analogues. Importantly, in the V analogues the vanadium ion is in the V(iii) oxidation state with a d^2^ configuration, however, the spin of the cluster remains the same. Hence upon going from a Mo to a V incorporated cubane, the lone spin-up electron from the Mo^3+^ ion has moved to the spin-down Fe, resulting in its reduction to Fe^2+^ (Fig. 2 right). To our knowledge, this assignment has not previously been made for FeVco, and there is no previous experimental data comparing the oxidation states or electronic structures of FeMoco and FeVco. While experimental validation of our DFT calculations is therefore difficult in the case of the cofactors, ample prior evidence exists to indicate that the DFT-calculated differences in the cubane cluster electronic structures are indeed accurate. A comparative study of the electronic structures of a series of synthetic [MoFe_3_S_4_]^3+^ and [VFe_3_S_4_]^2+^ clusters was performed by Carney *et al.*,^35^ in which M^3+^ oxidation states were found for both M = Mo and V. Furthermore, the iron complement of the vanadium clusters was found to be more reduced on the basis of zero-field ^57^Fe Mössbauer spectroscopy, consistent with the present DFT calculations. Finally, applied-field ^57^Fe Mössbauer spectroscopy of the synthetic cubane models allowed the authors to arrive at the identical spin coupling arrangement presented herein, and they noted “Two inequivalent subsites with a 2 : 1 intensity ratio … in which the magnetic moments of the Fe atoms of the more and less intense subsites are parallel and antiparallel, respectively”.^35^

The work of Carney *et al.* thus provides excellent verification of our DFT-calculated differences between the molybdenum and vanadium cubane cluster models. An additional important point obtained from their work is that the identity of the alternate ligand(s) on the heterometal has a negligible impact on the electronic structure of the MFe_3_S_4_ cluster core; thus the Tp and DMF ligands on the Mo and V clusters respectively, or the homocitrate/His motif on FeMoco (Fig. 1), do not engender any significant differences in the electronic structure.

The synthetic cubane clusters faithfully reproduce a key structural difference between the M-clusters: the iron–heterometal bond lengths. Based on periodic trends, and Shannon ionic radii,^36^ the larger Mo^3+^ ion should have longer bond lengths than V^3+^. Experimentally however, the opposite trend is observed. The high-resolution X-ray crystal structures of the cubane clusters reveal average Mo–Fe distances of 2.73 Å and V–Fe distances of 2.78 Å.^18,19^ The corresponding Mo–Fe distances in FeMoco are 2.69 Å, and the average V–Fe distances in FeVco (as determined by vanadium EXAFS) are 2.76 Å.^37,38^

The electronic structure calculations herein accurately reproduce these changes in metrical parameters, and were further analyzed with regard to the iron–heterometal interactions. Similar to our previous work,^4^ the Pipek–Mezey localized orbital populations were calculated for the iron–heterometal bonding-type orbitals, and the Mayer bond orders and Mulliken spin populations were examined (Fig. S5 and S6 and Table S3†). While we note that the exact numerical values are functional dependent, the Mo–Fe interactions consistently have more bonding character than the V–Fe interactions. This is consistent with the observed changes in metrical parameters, and can be rationalized by considering both the more diffuse d-orbitals of molybdenum (*vs.* vanadium, where contracted orbitals diminish overlap) as well as the more reduced iron complement of the vanadium clusters, where (as shown in Fig. 2) a formally covalent Mo–Fe bond becomes a dative V–Fe bond.

These results suggest that distinct differences in iron oxidation state as well as iron–heterometal interactions exist between the molybdenum- and vanadium-containing clusters. While there is experimental support for these findings in the case of the cubane cluster models,^35^ the validity of extending this insight to the FeMoco and FeVco active sites remains to be established. The X-ray spectroscopic experiments detailed herein were performed to investigate whether these electronic structural differences persist between the enzyme cofactors, and if they can rationalize the differences in reactivity discussed above.

### Fe Kα HERFD XAS

**Fig. 3:**
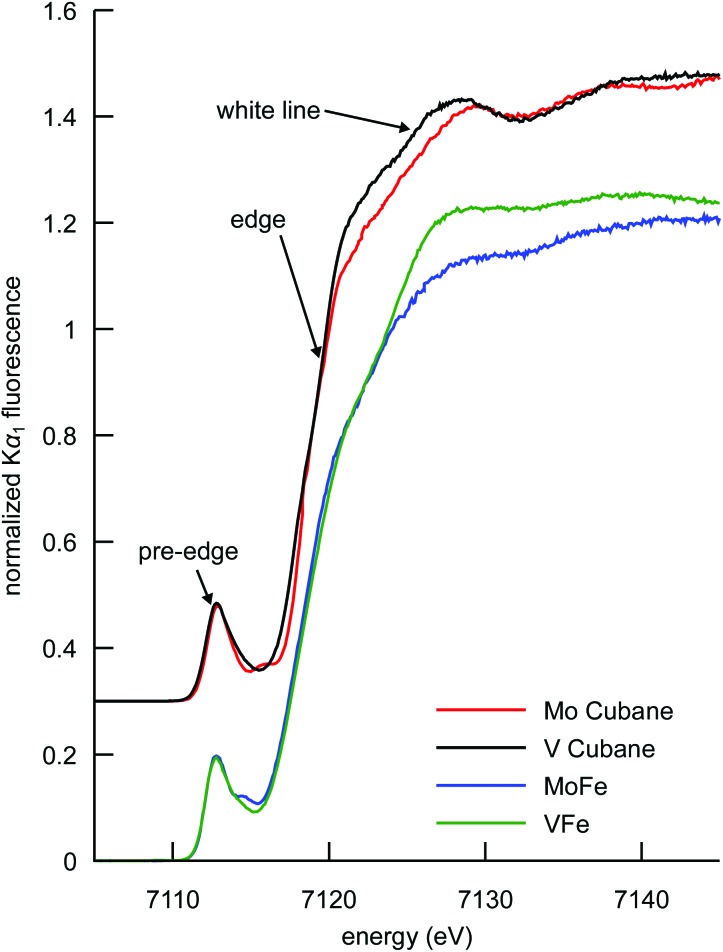
Comparison of the Fe Kα-detected HERFD XAS spectra of the synthetic MoFe_3_S_4_ and VFe_3_S_4_ cubane clusters, and the MoFe and VFe proteins. Cubane spectra have been vertically offset by 0.3 units for clarity.

The Fe Kα HERFD XAS spectra of the MoFe and VFe proteins and the synthetic [MoFe_3_S_4_]^3+^ and [VFe_3_S_4_]^2+^ cubane clusters are shown in Fig. 3. Typical of iron–sulfur clusters,^39^ the spectra exhibit a rising edge inflection at ∼7120 eV, a white line maximum at ∼7125 eV, and a pre-edge feature at ∼7113 eV, as determined from the first derivatives of the spectra (Fig. S3†). While the determination of metal ion oxidation state is a fundamental utility of XAS in bioinorganic chemistry, the pre-edge features and edge energy (as determined by the first inflection point of the rising edge) have been shown to be insensitive to oxidation state changes in iron–sulfur clusters with a localized “trapped valence” electronic structure.^40,41^ It is possible that the soft sulfur ligands compensate for changes in electron density at the metal *via* changes in covalency.^42^ Interestingly, however, the intensity of the white line region serves as an indicator of overall cluster oxidation state. A decrease in white line intensity in the XAS spectra of structurally analogous iron–sulfur clusters corresponds to a more oxidized cluster, as evidenced by many literature examples of both biological and synthetic iron–sulfur clusters.^39–41,43–46^

This interpretation clearly holds for the synthetic cubane cluster models in the present study; the more reduced iron complement of the [VFe_3_S_4_]^2+^ cubane (as previously determined by ^57^Fe Mössbauer spectroscopy, *vide supra*) manifests as a more intense white line compared to that of the [MoFe_3_S_4_]^3+^ cubane. In the case of the proteins, VFe also has a more intense white line than MoFe, indicating that the overall amount of ferrous iron in VFe is greater than in MoFe. The interpretation, however, is complicated by the presence of the P-clusters in addition to the M-clusters. In the MoFe protein, the P-cluster is in an all-ferrous state, while in the VFe protein it has been proposed on the basis of electron paramagnetic resonance (EPR) spectroscopy that the P-cluster could be one electron more oxidized.^47–49^ A more oxidized P-cluster contribution would tend to decrease the intensity of the white line in VFe relative to MoFe. We note that this phenomenon has been previously observed upon oxidation of the MoFe P-clusters.^45^ The fact that VFe has a more *intense* white line however suggests that the M-cluster in VFe is more reduced, regardless of the oxidation state of the P-cluster. Thus, the present data show that the total iron complement of FeVco is more reduced than FeMoco, consistent with the DFT calculations discussed above.

**Fig. 4:**
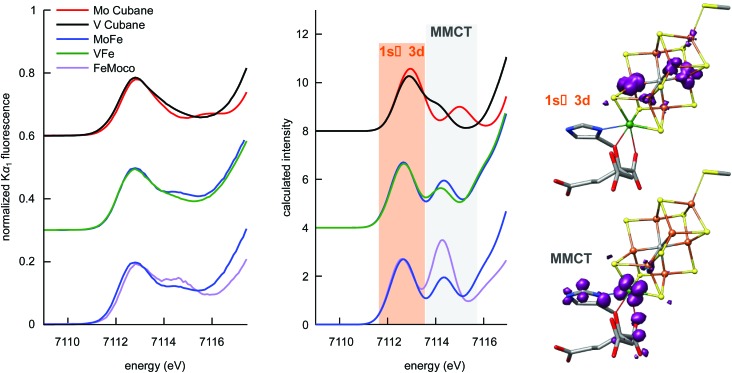
Comparison of the pre-edge regions of experimental Fe Kα-detected HERFD XAS (left) and TD-DFT-calculated XAS (middle) spectra of the synthetic MoFe_3_S_4_ and VFe_3_S_4_ cubane clusters, MoFe, VFe, and isolated FeMoco. Experimental data are offset in intervals of 0.3 units and calculated spectra in intervals of 4 units. A scalar shift of 122.1 eV and Gaussian broadening of 1.5 eV was added to the calculated transition energies to align theory with experiment. Right: TD-DFT-calculated transition difference densities for selected 1s → 3d and metal-to-metal charge transfer transitions at the indicated peaks. The purple isosurface is a positive density difference (acceptor), shown at a contour level of 0.004 a_0_^–3^ for FeMoco (analogous transitions are found for FeVco and the cubane models, and the negative density difference, or donor, is the highly contracted 1s, which is not visible).

At ∼7113 eV, the 1s → 3d pre-edge feature is observed (Fig. 4). These transitions are formally dipole forbidden, but gain intensity due to the symmetry-allowed 3p–4d mixing in the local ∼T^d^ symmetry.^46,50,51^ Closer inspection of the pre-edge region in Fig. 4 (left) shows the presence of additional features to higher energy at ∼7115 eV. For both the proteins and the synthetic cubane clusters, this second feature is more pronounced in the case of the molybdenum clusters, whereas in the respective vanadium analogues it appears as a high-energy shoulder. In addition, when comparing MoFe to the isolated FeMoco, the latter has greater intensity at the ∼7115 eV feature, which suggests that FeMoco (rather than the P-cluster) makes the primary contribution to this additional pre-edge feature. The persistence of the additional high-energy pre-edge features in the spectra of the synthetic cubane clusters (and the absence of any similar feature in iron sulfur model complexes)^41,46^ further implies that this feature occurs due to the presence of the heterometal.

We note that in addition to 1s → 3d transitions, pre-edge transitions can also arise due to metal-to-ligand charge transfer (MLCT) transitions, particularly when ligands possess low-lying unoccupied orbitals, *e.g.* an extended π* system.^52–54^ For iron–sulfur clusters with weak-field sulfide ligands and tetrahedral geometry however, a small Δ_T_ and the absence of MLCT-accepting ligands generally precludes observation of more than one pre-edge peak; accordingly, multiple pre-edge peaks are atypical for iron–sulfur clusters. As shown in Fig. 2 however, the Mo^3+^ and V^3+^ ions have unoccupied 4d and 3d orbitals respectively, which meet both the low-lying and unoccupied criteria for CT transitions. Hence empirically, it is possible that this feature may arise from metal-to-metal charge transfer (MMCT) transitions.

To further investigate the perturbations in the pre-edge region, TD-DFT calculations were performed. The calculated spectra are shown in Fig. 4 (center), and each trace is comprised of contributions from all iron atoms found in the respective samples. Importantly, this means that trends in the differences between calculated spectra, which accurately reproduce the experimental data, are representative of changes in electronic structure for all iron atoms in the sample. We note that in light of the potentially more oxidized P-cluster in VFe (*vide supra*), TD-DFT-calculated spectra were prepared for varying oxidation states of the P-cluster. As shown in Fig. S7,† variations in P-cluster oxidation state do not substantively alter the pre-edge region compared to the changes engendered by the identity of the heterometal. Thus, we can confidently attribute the observed differences between MoFe and VFe to differences in the M-clusters, rather than the P-clusters.

Having identified the origin of the spectral differences between MoFe and VFe as arising from the cofactors, the nature of the underlying transitions was then examined using the transition difference densities. As surmised from the empirical assessment above, the lower-energy pre-edge peak is found to correspond to transitions into Fe 3d orbitals, while the higher energy feature arises from MMCT transitions into the heterometal e_g_ orbitals (Fig. 4 right). It is noted that while some transitions to the heterometal t_2g_ orbitals can be found at ∼7113 eV (and likewise some 1s → Fe 3d transitions occur at ∼7115 eV), these are calculated to have minimal contributions to spectral intensity. The intensity of the MMCT feature is correlated to heterometal identity in both the cubane cluster models as well as the protein M-clusters. The electric-dipole-allowed MMCT transitions in the pre-edge region have an intensity that is proportional to the overlap of the donor (Fe 1s) and acceptor (heterometal e_g_) wavefunctions, implying mixing of the molybdenum and vanadium 4d and 3d orbitals, respectively, with the iron-based valence orbitals. The diminished intensity in the case of the vanadium analogues indicates less V/Fe orbital mixing. This could be due in part to the less diffuse 3d valence orbitals of vanadium compared to the 4d valence shell of molybdenum. However, changes in metrical parameters and the weaker V–Fe bonding (compared to Mo–Fe) evidenced by our DFT calculations likely contribute as well. As a final note, while one may expect a smaller overall change to the MMCT feature in the protein spectra given the larger number of total iron atoms in the sample, the iron–heterometal bond lengths in the cofactors are shorter than in the respective synthetic MFe_3_S_4_ cubane clusters, which increases the overall intensity and emphasizes any differences in this spectral region.

With regard to metrical parameters, distribution of oxidation states, and heterometal bonding, the synthetic cubane clusters constitute effective models for the heterometal-induced changes to cofactor electronic structure. However, the synthetic cubane clusters do not contain the unique interstitial carbide found at the center of the cofactors.^3,12^ Thus, a complementary spectroscopic comparison of the enzyme cofactors to the synthetic cubane models may provide some insight into the perturbations engendered by the interstitial carbide.

### Fe Kβ XES

The Kβ mainline region of an XES spectrum corresponds to the fluorescence that results when a 3p electron fills a 1s core hole (in the present case on an iron atom) following photoionization. The final state of the 3p → 1s fluorescence contains an unpaired electron in the 3p shell, which can exchange couple to unpaired electrons of the same spin in the 3d orbitals.^55–58^ This has resulted in the frequent use of Kβ mainlines as a probe of oxidation and spin state. However, we have recently shown that Kβ mainline spectra can also serve as an experimental probe of covalency.^59^ Specifically, the covalent delocalization of 3d spin population decreases the magnitude of the 3p–3d exchange coupling. This manifests in the Kβ XES spectrum as a decrease in the energetic splitting of Kβ′ and Kβ_1,3_ spectral features, termed Δ*E*_main_. Of particular interest in the context of the present study is the fact that for covalent iron-sulfur clusters, it has been shown that the decrease in spin state and decrease in covalency upon reduction exactly cancel each other. This results in iron–sulfur clusters of different oxidation states having fully superimposable Kβ mainlines.^41^

**Fig. 5:**
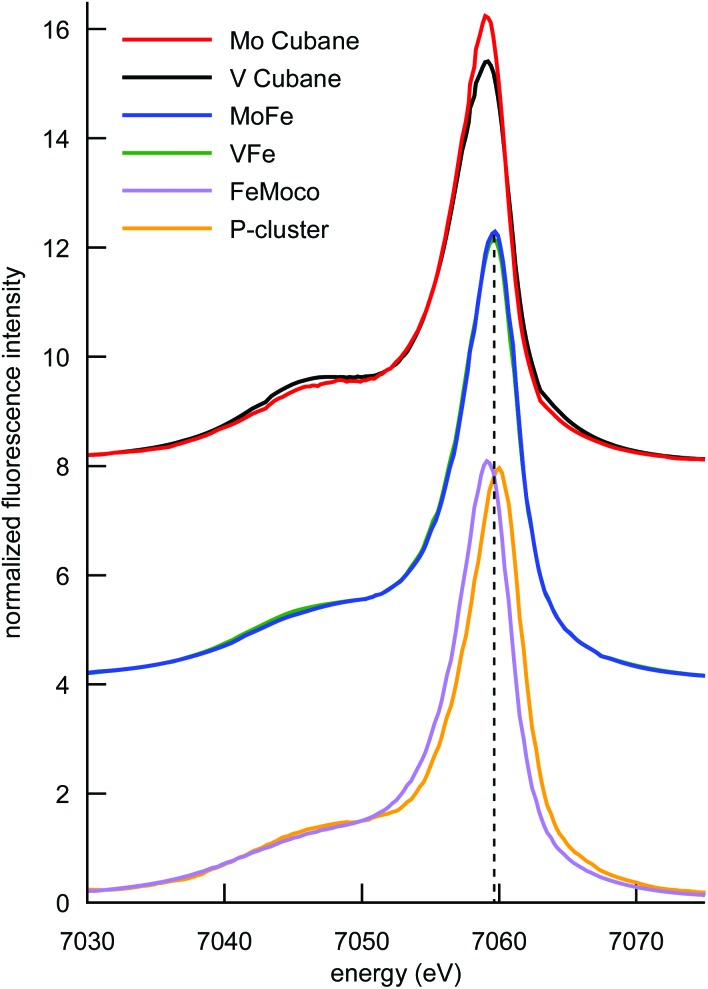
Comparison of the Fe Kβ mainlines of the synthetic MoFe_3_S_4_ and VFe_3_S_4_ cubane clusters, MoFe, VFe, FeMoco, and the P-cluster. Spectra have been vertically offset in intervals of 4 units for clarity. The vertical dashed line is placed at the Kβ_1,3_ peak position for the MoFe and VFe, which falls between the respective peak positions of FeMoco and the P-cluster.

The Kβ mainline XES spectra of the cubane cluster models, the MoFe and VFe proteins, and isolated FeMoco and the P-cluster-only variant of MoFe are shown in Fig. 5. Perturbations to the relative spectral intensities are observed for the MoFe_3_S_4_ and VFe_3_S_4_ cubane clusters, and they have effectively identical Kβ mainline splitting, with a Kβ_1,3_ feature at ∼7058 eV and a Kβ′ at ∼7048 eV (Table S2†). This is despite the known differences in the iron oxidation states; thus, this observation is consistent with previous reports on Fe_2_S_2_ dimers^41,46^ and provides further evidence for the relative insensitivity of Kβ mainline splittings to changes in oxidation state in highly covalent iron–sulfur clusters.

Comparison of the Kβ mainline spectra for the MoFe and VFe proteins shows that like the models, the two proteins also have effectively identical Kβ mainline spectra, with the Δ*E*_main_ splittings decreasing to only ∼9.5 eV (compared to ∼10 eV in the case of the cubane cluster models). As noted above, these spectra will have contributions from both the M-cluster and the P-cluster; hence it is of utility to examine the Kβ mainline splittings of isolated FeMoco and a P-cluster-only variant of MoFe in order to understand the relative contributions. It is seen that the P-cluster-only variant has a Δ*E*_main_ of ∼11 eV, while isolated FeMoco has a Δ*E*_main_ of 9.1 eV. This suggests that the dominant contributions to the reduced Kβ mainline splitting in MoFe (and by inference in VFe) may be attributed to the M-clusters. Further, we note that to our knowledge, Δ*E*_main_ values <11 eV have never been reported for any high-spin ferrous or ferric systems. Hence, the present results highlight the unique electronic structural characteristic of both the synthetic cubane clusters and the M-clusters relative to other iron sulfur clusters. In the case of the cubane clusters, the modulation of the splitting may be attributed to the presence of the heterometal. The fact that the Kβ mainline splitting is even smaller for the MoFe and VFe proteins (and smallest for isolated FeMoco) suggests that the highly covalent carbide likely plays a further role in modulating the electronic structure.^60^

### Spin-polarized Fe Kβ HERFD XAS

**Fig. 6:**
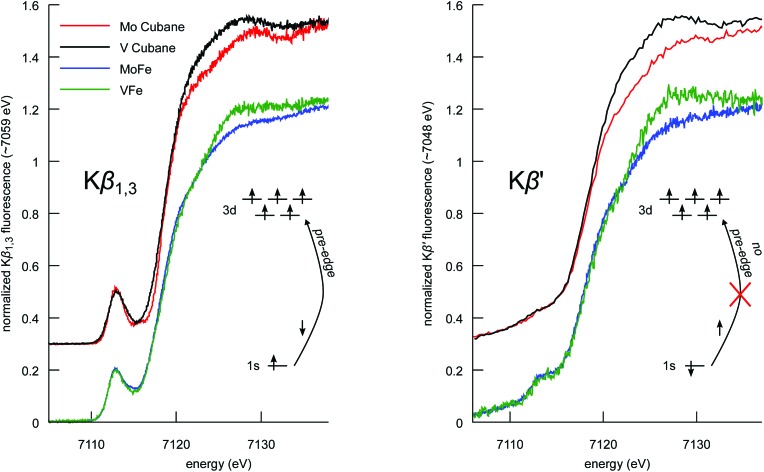
Comparison of the Fe Kβ_1,3_- (left) and Kβ′-detected (right) HERFD XAS spectra of the synthetic MoFe_3_S_4_ and VFe_3_S_4_ cubane clusters, and MoFe and VFe proteins. Cubane spectra have been vertically offset by 0.3 for clarity. In a one-electron picture, the schematics illustrate how spin selectivity results in a loss of 1s → 3d transitions.

The Kβ_1,3_ and Kβ′ spectral features arise from different final state spin multiplets: the ^7^P and ^5^P in the case of a high-spin d^5^ ion.^57,59^ As these multiplets correspond to opposite spin polarizations in the 3p^5^ final state, Kβ HERFD XAS offers spin-selective detection channels.^61,62^ Fig. 6 shows the Kβ HERFD XAS spectra of the MFe_3_S_4_ cubane cluster models, MoFe, and VFe. The Kβ_1,3_-detected spectra (left) have a strong resemblance to the Kα HERFD spectra in Fig. 3. In a one-electron picture, these transitions are largely confined to the spin-down manifold, and the spin-allowed 1s → 3d excitations give rise to strong pre-edge features. In contrast, the Kβ′ detection channel (Fig. 6 right) results in suppression of the pre-edge features. A high-spin d^5^ configuration has no spin-allowed 1s → 3d transitions in the spin-up transition manifold, and thus minimal pre-edge features are observed. We note, however, that there is greater pre-edge intensity for the proteins relative to the synthetic cubane clusters in the Kβ′-detected spectra. This likely results from the compression of the spin multiplets in the M-cluster spectra, as also observed in the Kβ mainline spectra. Importantly, these data provide clear experimental evidence that the electronic structures of the protein cofactors are distinct from those of the synthetic cubane clusters. Finally, we note that the differences in white line intensity in the Kα HERFD spectra also persist in both sets of Kβ HERFD data.

## Discussion

The spectroscopic data and computational results have yielded new insight into the active site cofactors of the nitrogenase enzymes, with the synthetic cubane clusters providing important reference data. Namely, the iron complement of FeVco is more reduced than FeMoco, giving rise to less covalent iron–ligand bonds in FeVco. Additionally, as evidenced by the relative intensities of the MMCT transitions, the iron–heterometal bonds are weaker for the vanadium-containing clusters. Finally, the presence of the interstitial carbide is found to impart a substantial increase in the overall covalency of the cofactors compared to other iron–sulfur clusters. While these distinctions are fundamentally interesting, their implications for the differential reactivity of the molybdenum and vanadium nitrogenases, as well as possible functional roles of the heterometal and interstitial carbide, warrant some discussion.

While the relative iron oxidation states of the cubane cluster models are known to differ by only one,^35^ the present data do not definitively establish the same relationship for the cofactors. In light of a potentially more oxidized VFe P-cluster, it is possible that the iron complement of FeVco is more reduced by three electrons (rather than one), in order to maintain the overall quartet ground state established by EPR spectroscopy.^1^ A more quantitative understanding of the exact level of reduction in FeVco is the subject of ongoing investigations.

Regardless of the exact number of ferrous ions present in FeVco, the fact that a more reduced iron complement is present relative to FeMoco has possible implications for reactivity, particularly with CO. Studies by Ribbe and coworkers have shown that the vanadium isozyme is capable of reducing CO to short-chain hydrocarbons,^10^ and that CO will bind to FeVco in the resting state.^13^ In contrast, FeMoco only coordinates CO once reduced under turnover conditions, and is reversibly inhibited by CO.^11^ Interestingly, X-ray crystallography^63^ has shown that CO coordinates to FeMoco in a bridging μ_2_ fashion, *via* displacement of the S2B “belt” sulfide ion; and EPR spectroscopy^13^ has been used to suggest a similar binding motif in FeVco. As a π-accepting ligand, CO binding is promoted by the increased π backbonding caused by the reduction of metal ions; thus the binding of CO to the resting state of VFe (but not MoFe) is fully consistent with a more reduced iron complement in FeVco.

In addition to the differential reactivity towards CO, the activity of the two nitrogenase isozymes for the native N_2_ reduction are markedly different (Table S1†). By all measures, the vanadium nitrogenase is a less active, less efficient catalyst that is more prone to degradation (as measured by the turnover number, TON). This is further evidenced by the preferential expression of the molybdenum nitrogenase at ambient temperatures.^1^ Based on the differences in electronic structure we have established above, a rationale for this trend can be proposed. It has been shown that upon turnover, the same S2B belt sulfide that is displaced by CO can be exchanged for a selenide.^64^ This implies that sulfide lability may be catalytically relevant for N_2_ reduction. While Varley *et al.* have proposed a series of bioenergetically-viable steps leading to S2B dissociation,^65^ Dance has reported a substantial DFT-calculated energy barrier to formation of a sulfide-deficient reaction intermediate (with 3-coordinate iron atoms).^66^ While the precise mechanism of substrate reduction remains an open question, it is clear that the enzyme must maintain the structural integrity of the cofactor in the absence of S2B, *e.g.* in the crystallographically-characterized CO-bound state.^63^ Thus, in FeMoco (compared to FeVco), the presence of more covalent iron–ligand bonds and stronger bonds to the heterometal may improve cofactor stability during turnover. As the interstitial carbide forms six highly covalent bonds to iron atoms, one might imagine that its role could be to function as a central anchor that holds together the cofactor during turnover.^6,60,66–68^ Likewise, the presence of iron–heterometal bonds could further suggest that the heterometal also contributes to maintaining the structural integrity of the cofactor.

## Conclusions

The results presented herein directly illustrate differences in electronic structure between FeMoco and FeVco. Through a combination of X-ray spectroscopic approaches, supported by electronic structure calculations, it has been demonstrated that a less covalent and more reduced iron complement is present in FeVco. In addition, the presence of iron–heterometal bonding has been spectroscopically established, with the Mo–Fe bonding contribution being greater than the V–Fe bonding contribution. These electronic structure differences may be important factors in governing the differential stability and activity of these enzymes. Furthermore, we have shown that the presence of the interstitial carboide in FeMoco and FeVco serves to increase the overall covalency of the cofactors, which may help maintain cofactor structural integrity during catalysis.

## References

[cit1] Eady R. (2003). Coord. Chem. Rev..

[cit2] Spatzal T., Aksoyoglu M., Zhang L., Andrade S. L. A., Schleicher E., Weber S., Rees D. C., Einsle O. (2011). Science.

[cit3] Lancaster K. M., Roemelt M., Ettenhuber P., Hu Y., Ribbe M. W., Neese F., Bergmann U., DeBeer S. (2011). Science.

[cit4] Bjornsson R., Lima F. A., Spatzal T., Weyhermüller T., Glatzel P., Bill E., Einsle O., Neese F., DeBeer S. (2014). Chem. Sci..

[cit5] Spatzal T., Einsle O., Andrade S. L. A. (2013). Angew. Chem., Int. Ed..

[cit6] Spatzal T., Schlesier J., Burger E.-M., Sippel D., Zhang L., Andrade S. L. A., Rees D. C., Einsle O. (2016). Nat. Commun..

[cit7] Bjornsson R., Neese F., DeBeer S. Inorg. Chem..

[cit8] Ravi N., Moore V., Lloyd S. G., Hales B. J., Huynh B. H. (1994). J. Biol. Chem..

[cit9] Miller R. W., Eady R. R. (1988). Biochem. J..

[cit10] Lee C. C., Hu Y., Ribbe M. W. (2010). Science.

[cit11] Burgess B. K. (1990). Chem. Rev..

[cit12] Rees J. A., Bjornsson R., Schlesier J., Sippel D., Einsle O., DeBeer S. (2015). Angew. Chem., Int. Ed..

[cit13] Lee C. C., Fay A. W., Weng T.-C., Krest C. M., Hedman B., Hodgson K. O., Hu Y., Ribbe M. W. (2015). Proc. Natl. Acad. Sci. U. S. A..

[cit14] Lancaster K. M., Hu Y., Bergmann U., Ribbe M. W., DeBeer S. (2013). J. Am. Chem. Soc..

[cit15] Sippel D., Schlesier J., Rohde M., Trncik C., Decamps L., Djurdjevic I., Spatzal T., Andrade S. L. A., Einsle O. (2017). J. Biol. Inorg. Chem..

[cit16] Shah V. K., Brill W. J. (1977). Proc. Natl. Acad. Sci. U. S. A..

[cit17] Shah V. K., Brill W. J. (1981). Proc. Natl. Acad. Sci. U. S. A..

[cit18] Fomitchev D. V., McLauchlan C. C., Holm R. H. (2002). Inorg. Chem..

[cit19] Kovacs J. A., Holm R. H. (1986). J. Am. Chem. Soc..

[cit20] Smolentsev G., Soldatov A. V., Messinger J., Merz K., Weyhermüller T., Bergmann U., Pushkar Y., Yano J., Yachandra V. K., Glatzel P. (2009). J. Am. Chem. Soc..

[cit21] Finkelstein K. D., Pollock C. J., Lyndaker A., Krawcyk T. and Conrad J., Dual-array valence emission spectrometer (DAVES): A new approach for hard x-ray photon-in photon-out spectroscopies, in AIP Conference Proceedings, ed. Q. Shen and C. Nelson, AIP Publishing, 2016, vol. 1741, No. 1, 10.1063/1.4952832.

[cit22] van Schooneveld M. M., DeBeer S. (2015). J. Electron Spectros. Relat. Phenomena.

[cit23] Solé V. A., Papillon E., Cotte M., Walter P., Susini J. (2007). Spectrochim. Acta, Part B.

[cit24] Stoll S., Schweiger A. (2006). J. Magn. Reson..

[cit25] Neese F. (2012). Wiley Interdiscip. Rev.: Comput. Mol. Sci..

[cit26] Becke A. (1988). Phys. Rev. A.

[cit27] Perdew J. P. (1986). Phys. Rev. B: Condens. Matter.

[cit28] Hess B. A. (1985). Phys. Rev. A: At., Mol., Opt. Phys..

[cit29] Hess B. A. (1986). Phys. Rev. A: At., Mol., Opt. Phys..

[cit30] Jansen G., Hess B. A. (1989). Phys. Rev. A: At., Mol., Opt. Phys..

[cit31] Pantazis D. A., Chen X. Y., Landis C. R., Neese F. (2008). J. Chem. Theory Comput..

[cit32] Weigend A. (2006). Phys. Chem. Chem. Phys..

[cit33] Klamt A., Schüürmann G. (1993). J. Chem. Soc., Perkin Trans. 2.

[cit34] DeBeer George S., Petrenko T., Neese F. (2008). J. Phys. Chem. A.

[cit35] Carney M. J., Kovacs J. A., Zhang Y. P., Papaefthymiou G. C., Spartalian K., Frankel R. B., Holm R. H. (1987). Inorg. Chem..

[cit36] Shannon R. D. (1976). Acta Crystallogr., Sect. A: Cryst. Phys., Diffr., Theor. Gen. Cryst..

[cit37] Arber J. M., Dobson B. R., Eady R. R., Hasnain S. S., Garner C. D., Matsushita T., Nomura M., Smith B. E. (1989). Biochem. J..

[cit38] George G. N., Coyle C. L., Hales B. J., Cramer S. P. (1988). J. Am. Chem. Soc..

[cit39] Kowalska J., DeBeer S. (2015). Biochim. Biophys. Acta, Mol. Cell Res..

[cit40] Musgrave K. B., Isaac H., Li L., Burgess B. K., Watt G., Hedman B., Hodgson K. O. (1998). J. Biol. Inorg. Chem..

[cit41] Kowalska J. K., Hahn A. W., Albers A., Schiewer C. E., Bjornsson R., Lima F. A., Meyer F., DeBeer S. (2016). Inorg. Chem..

[cit42] Lugo-Mas P., Dey A., Xu L., Davin S. D., Benedict J., Kaminsky W., Hodgson K. O., Hedman B., Solomon E. I., Kovacs J. A. (2006). J. Am. Chem. Soc..

[cit43] Musgrave K. B., Angove H. C., Burgess B. K., Hedman B., Hodgson K. O. (1998). J. Am. Chem. Soc..

[cit44] Corbett M. C., Hu Y., Fay A. W., Ribbe M. W., Hedman B., Hodgson K. O. (2006). Proc. Natl. Acad. Sci. U. S. A..

[cit45] Corbett M. C., Hu Y., Naderi F., Ribbe M. W., Hedman B., Hodgson K. O. (2004). J. Biol. Chem..

[cit46] Yao S., Meier F., Lindenmaier N., Rudolph R., Blom B., Adelhardt M., Sutter J., Mebs S., Haumann M., Meyer K., Kaupp M., Driess M. (2015). Angew. Chem., Int. Ed..

[cit47] Lee C. C., Hu Y., Ribbe M. W. (2009). Proc. Natl. Acad. Sci. U. S. A..

[cit48] Morningstar J. E., Johnson M. K., Case E. E., Hales B. J. (1987). Biochemistry.

[cit49] Hu Y., Corbett M. C., Fay A. W., Webber J. A., Hedman B., Hodgson K. O., Ribbe M. W. (2005). Proc. Natl. Acad. Sci. U. S. A..

[cit50] Shulman G. R., Yafet Y., Eisenberger P., Blumberg W. E. (1976). Proc. Natl. Acad. Sci. U. S. A..

[cit51] Westre T. E., Kennepohl P., DeWitt J. G., Hedman B., Hodgson K. O., Solomon E. I. (1997). J. Am. Chem. Soc..

[cit52] Rees J. A., Martin-Diaconescu V., Kovacs J. A., DeBeer S. (2015). Inorg. Chem..

[cit53] Roemelt M., Beckwith M. A., Duboc C., Collomb M.-N., Neese F., DeBeer S. (2012). Inorg. Chem..

[cit54] Leto D. F., Jackson T. A. (2014). Inorg. Chem..

[cit55] Glatzel P., Bergmann U. (2005). Coord. Chem. Rev..

[cit56] Pollock C. J., DeBeer S. (2015). Acc. Chem. Res..

[cit57] Kowalska J. K., Lima F. A., Pollock C. J., Rees J. A., DeBeer S. (2016). Isr. J. Chem..

[cit58] Bergmann U., Glatzel P. (2009). Photosynth. Res..

[cit59] Pollock C. J., Delgado-Jaime M. U., Atanasov M., Neese F., DeBeer S. (2014). J. Am. Chem. Soc..

[cit60] Pollock C. J., Tan L. L., Zhang W., Lancaster K. M., Lee S. C., DeBeer S. (2014). Inorg. Chem..

[cit61] Wang X., Randall C. R., Peng G., Cramer S. P. (1995). Chem. Phys. Lett..

[cit62] Wang X., de Groot F., Cramer S. (1997). Phys. Rev. B: Condens. Matter.

[cit63] Spatzal T., Perez K. A., Einsle O., Howard J. B., Rees D. C. (2014). Science.

[cit64] Spatzal T., Perez K. A., Howard J. B., Rees D. C. (2015). eLife.

[cit65] Varley J. B., Wang Y., Chan K., Studt F., Nørskov J. K. (2015). Phys. Chem. Chem. Phys..

[cit66] Dance I. (2016). Dalton Trans..

[cit67] Wiig J. A., Lee C. C., Hu Y., Ribbe M. W. (2013). J. Am. Chem. Soc..

[cit68] Siegbahn P. E. M. (2016). J. Am. Chem. Soc..

