# Supplementary material for: Comparative electronic structures of nitrogenase FeMoco and FeVco
Source: Dalton Trans. 2017 Feb 2;46(8):2445–55. doi: 10.1039/c7dt00128b (PMC5322470; doi:10.1039/c7dt00128b)
Supplement: Supplementary file 1 [file DT-046-C7DT00128B-s001.pdf]

Electronic Supplementary Material (ESI) for Dalton Transactions.
This journal is © The Royal Society of Chemistry 2017

Supplementary Information for

Comparative Electronic Structures of Nitrogenase FeMoco and FeVco

Julian A. Rees,[a,b] Ragnar Bjornsson,[a,c] Joanna K. Kowalska,[a] Frederico A. Lima,[a,d] Julia Schlesier,[e] Daniel Sippel,[e] Thomas Weyhermüller,[a] Oliver Einsle,[e]* Julie A. Kovacs[b]* and Serena DeBeer[a,f]*

[a]Max Planck Institute for Chemical Energy Conversion, Stiftstr. 34-36, 45470 Mülheim an der Ruhr, Germany, E-mail: serena.debeer@cec.mpg.de; Tel:+49 208 306 3605
[b]Department of Chemistry, University of Washington, Box 351700, Seattle, WA 98195-1700, United States
[c]Science Institute, University of Iceland, Dunhagi 3, 107 Reykjavik, Iceland
[d]Centro Nacional de Pesquisa em Energia e Materiais Brazilian Synchrotron Light Laboratory - LNLS Rua Giuseppe Máximo Scolfaro, 10.000 13083-970 Campinas SP, Brazil
[e]Institute for Biochemistry and BIOSS Centre for Biological Signalling Studies, Albert Ludwigs University Freiburg, Germany
[f]Department of Chemistry and Chemical Biology, Cornell University, Ithaca, NY 14853, United States

List of Figures

S1 – Kβ mainline XES spectra and first derivatives of Fe_2O_3
S2 – Full spectral window of Kα HERFD XAS spectra, with post-edge for normalization
S3 – First derivatives of Kα HERFD XAS spectra
S4 – First derivatives of Kβ mainline XES spectra
S5 – DFT-calculated localized orbitals of cubane cluster models
S6 – DFT-calculated localized orbitals of protein cofactors
S7 – DFT-calculated XAS spectra of proteins with varying oxidation states of VFe P-cluster

List of Tables

S1 – Activity of nitrogenase isozymes
S2 – Energies of Kβ mainline XES features
S3 – Calculated spin populations and bonding analyses

Optimized Coordinates

$[VFe_3S_4Cl_3(DMF)_3]^-$

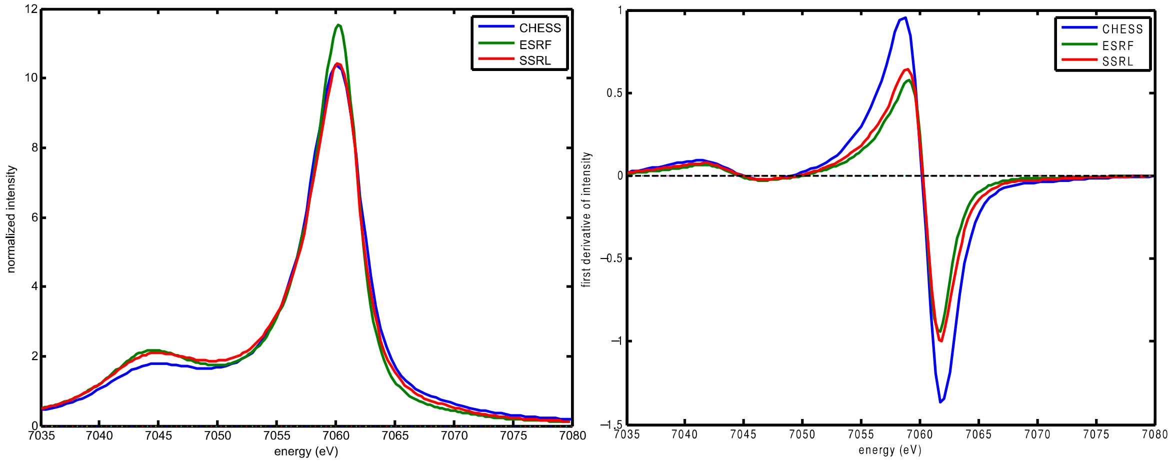

Fig. S1 Kβ mainline XES spectra (left) and first derivatives (right) of the Fe_2O_3 calibrant from three different synchrotron beamlines, showing identical peak positions.

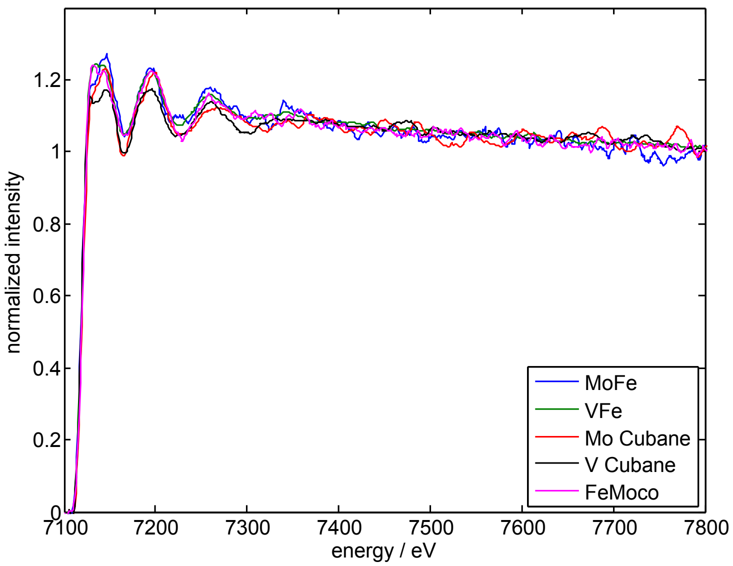

Fig. S2 Fe Kα-detected HERFD XAS spectra of the $MoFe_3S_3$ and VFe_3S_4 cubane clusters, MoFe, VFe, and FeMoco. The large spectral window includes the EXAFS region, which was normalized to a final intensity of 1.

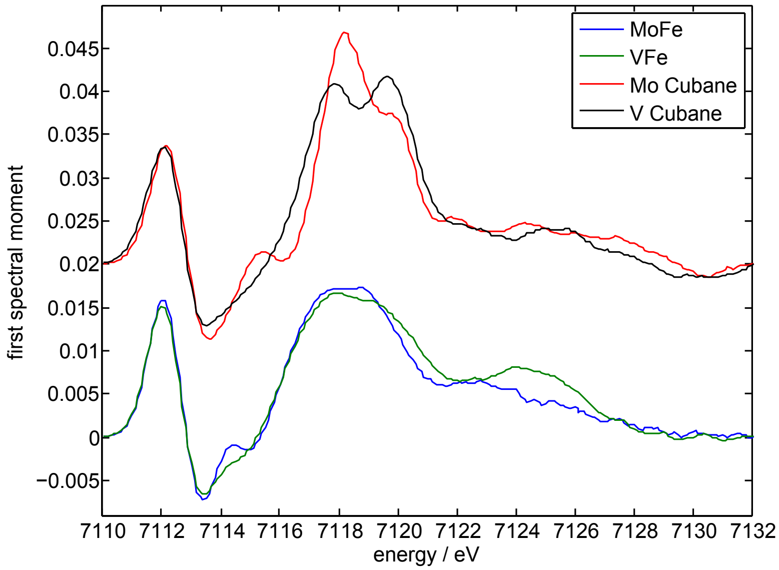

Fig. S3 Smoothed first spectral moments of the Fe Kα-detected HERFD XAS spectra of the $MoFe_3S_3$ and VFe_3S_4 cubane clusters, MoFe, and VFe. Cubane spectra have been vertically offset by 0.02.

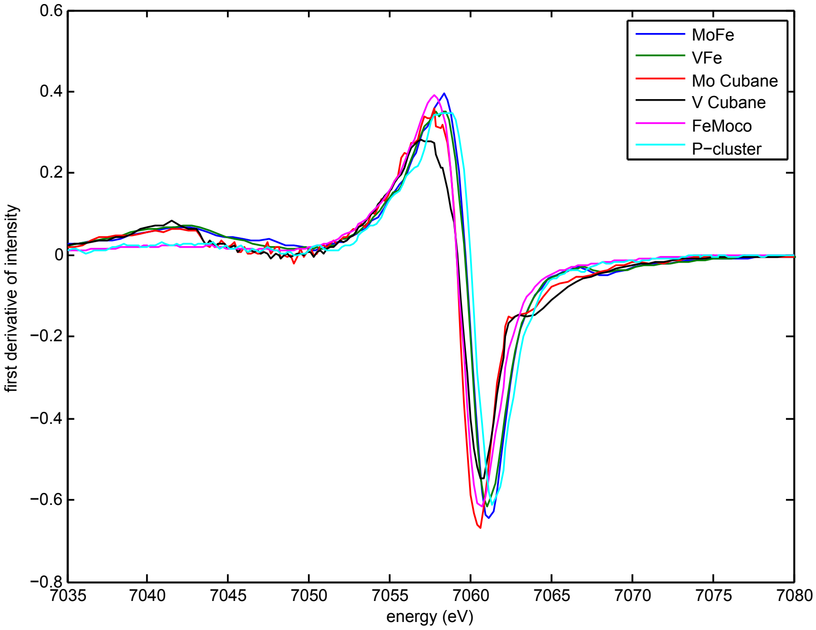

Fig. S4 Smoothed first spectral moments of the mainline region of the Fe Kβ XES spectra of the $MoFe_3S_3$ and VFe_3S_4 cubane clusters, MoFe, VFe, FeMoco, and the P-cluster-only MoFe variant.

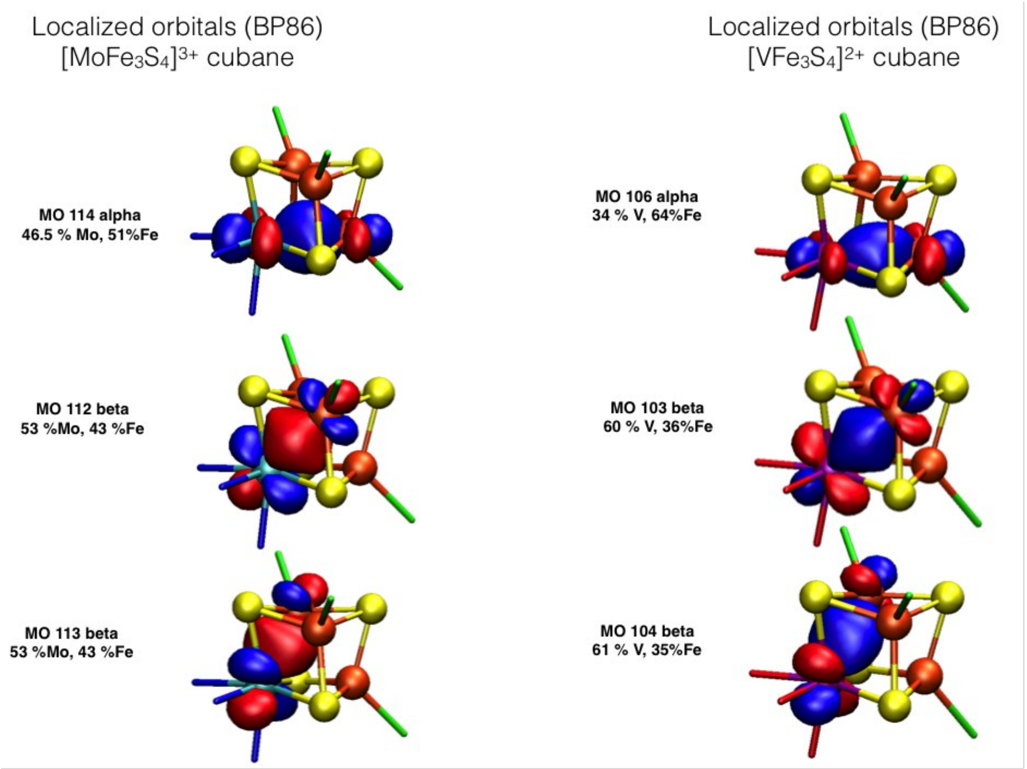

Fig. S5 Pipek-Mezey localized heterometallic bonding orbitals of the $[MoFe_3S_4]^{3+}$ and $[VFe_3S_4]^{2+}$ model cubanes

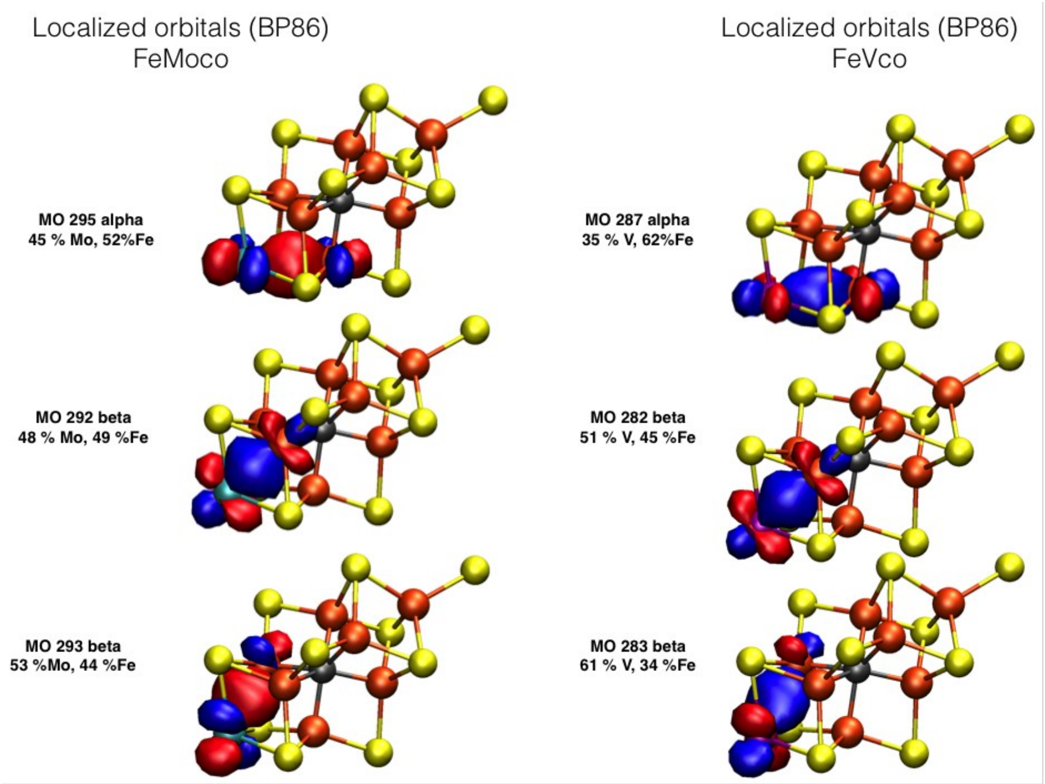

Fig. S6 Pipek-Mezey localized heterometallic bonding orbitals of FeMoco and FeVco

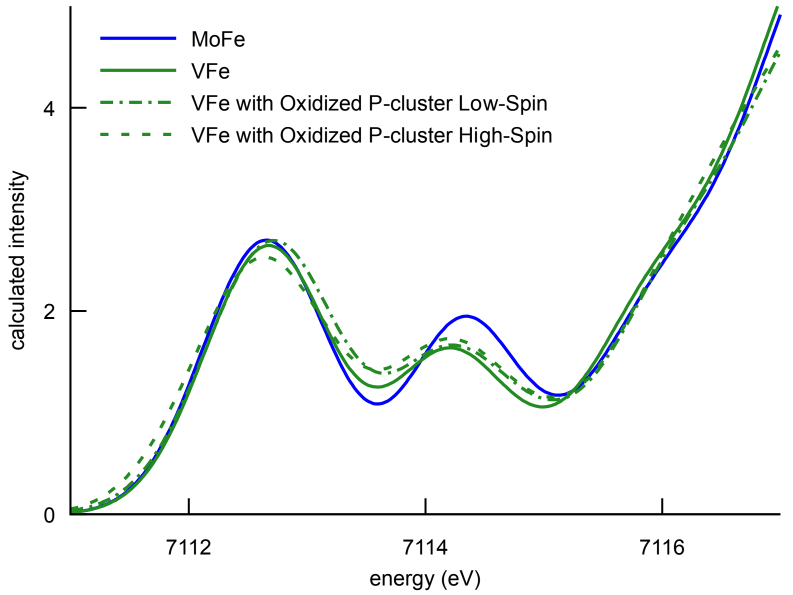

Fig. S7 TD-DFT-calculated XAS spectra of MoFe, VFe, and VFe with a one-electron oxidized P-cluster. For both the high-spin ($S = {}^5/_2$) and low-spin ($S = {}^1/_2$) cases, the changes to the second pre-edge peak are small compared to the perturbation due to a changing heterometal (VFe to MoFe). Also, changes to the first pre-edge peak are expected, in contrast to the experimental data.

Table S1 Literature values for enzyme activities of *A. vinelandii* molybdenum and vanadium nitrogenase. Values for usage and production during N_2 reduction are in molar equivalents per N_2, and approximate activities for CO reduction are reported as molar equivalents per protein per minute.[1–4]

N_2	Mo Nitrogenase	V Nitrogenase
MgATP Used	16	40
e^- used	8	12
H_2 produced	1	3[a]
Activity	1040	660
TON	2230	112
CO (eq. / min)		
C_2H_4	0.006	7.5
C_2H_6	0.001	2.5
C_3H_8	0.002	1.5

[a] It is noted that while this value has not been contradicted in the literature, a pressure-dependent study analogous to that of Simpson and Burris[1] has not been performed for the vanadium nitrogenase. Thus, we caution that this number is not as firmly established as the analogous H_2 production stoichiometry for the molybdenum isozyme.

Table S2 Energies of the Kβ mainline XES spectral features as determined from the first spectral moments. The Fe_2S_2 complexes were previously reported.[5]

	Kβ'	$K\beta_{1,3}$	ΔE_{main}
Mo Cubane	7048.0	7058.1	10.1
V Cubane	7048.0	7058.2	10.2
MoFe	7050.2	7059.7	9.5
VFe	7050.2	7059.7	9.5
FeMoco	7049.0	7058.1	9.1
P-cluster	7049.0	7060.0	11
Fe_2S_2 Complexes			
$Fe(III)_2$			13.4
Fe(II,III)			13.4
$Fe(II)_2$			13.4

Table S3 Mulliken spin population and Mayer bond order analyses from the DFT calculations (BP86) for Mo and V cubanes, FeMoco, and FeVco

Cubane Cluster Models				Protein Cofactors			
$[MoFe_3S_4]^{3+}$		$[VFe_3S_4]^{2+}$		$[MoFe_7S_9C]^{1-}$		$[VFe_7S_9C]^{2-}$	
Mulliken spin populations				Mulliken spin populations			
Mo	-0.22	V	-0.82	Mo	-0.13	V	-0.68
Fe1	-2.78	Fe1	-3.04	Fe1	+3.02	Fe1	+3.12
Fe2	+2.82	Fe2	+3.14	Fe2	+2.72	Fe2	+2.84
Fe3	+2.82	Fe3	+3.14	Fe3	-2.47	Fe3	-2.70
				Fe4	-2.35	Fe4	-2.61
				Fe5	-2.31	Fe5	-2.59
				Fe6	+1.97	Fe6	+2.41
				Fe7	+2.00	Fe7	+2.53
Mayer bond orders				Mayer bond orders			
Mo – Fe1	0.486	V – Fe1	0.447	Mo – Fe5	0.490	V – Fe5	0.448
Mo – Fe2	0.514	V – Fe2	0.480	Mo – Fe6	0.537	V – Fe6	0.470
Mo – Fe3	0.415	V – Fe3	0.480	Mo – Fe7	0.497	V – Fe7	0.393

Optimized Coordinates

The DFT-optimized coordinates for the 225-atom model of MoFe, the P-cluster, and the Mo cubane cluster are available in the SI from Bjornsson et al.[6] The optimized coordinates of the 225-atom model of VFe can found in the SI of Rees et al.[7] The optimized coordinates for the $[VFe_3S_4Cl_3(DMF)_3]^-$ V cubane cluster are below:

V	9.455748	-0.457301	2.149351
Fe	10.940228	1.743053	2.551326
Fe	8.472185	1.628859	3.415400
Fe	8.953574	1.772873	0.845867
S	10.161632	0.401666	4.165883
S	7.381389	0.447276	1.810355
S	9.486292	3.383944	2.369049
S	10.823996	0.600084	0.628039
Cl	13.096822	2.134259	2.978258
Cl	6.977963	2.042243	5.043279
Cl	8.142751	2.397494	-1.154520
O	11.021112	-1.942063	2.360513
C	12.206772	-1.623855	2.598005
N	13.201656	-2.511631	2.730035
C	14.558584	-2.062890	3.006218
C	12.964020	-3.941399	2.601853
O	8.474392	-2.026464	3.270923
C	7.640625	-1.756550	4.164332
N	7.020540	-2.683321	4.905457
C	6.065605	-2.288701	5.931132
C	7.276897	-4.103357	4.716967
O	8.981109	-1.877049	0.585783
C	8.522405	-1.495402	-0.514198
N	8.234212	-2.323632	-1.525993
C	7.708239	-1.801358	-2.779074
C	8.437701	-3.760153	-1.413397
H	12.518174	-0.572355	2.716921
H	15.237580	-2.383329	2.201714
H	14.913798	-2.484530	3.958560
H	14.570943	-0.968058	3.071453
H	13.566727	-4.351644	1.777721
H	13.243435	-4.455463	3.533741
H	11.900176	-4.098181	2.396590
H	7.359609	-0.717591	4.405493
H	6.398843	-2.643058	6.918075
H	5.986974	-1.194902	5.950158
H	5.075501	-2.717736	5.715817
H	7.662897	-4.543201	5.648657
H	8.016663	-4.219501	3.918424
H	6.346191	-4.621082	4.440633
H	8.324720	-0.431178	-0.725804
H	6.717396	-2.232730	-2.986029
H	7.617728	-0.710745	-2.706587
H	8.384911	-2.052041	-3.609729
H	7.483644	-4.288390	-1.559258
H	8.833164	-3.977721	-0.416061
H	9.150952	-4.100827	-2.178792

(1) Simpson, F. B.; Burris, R. H. *Sci. (New York, NY)* **1984**, *224*, 1095–1097.
(2) Burgess, B. K. *Chem. Rev.* **1990**, *90*, 1377–1406.
(3) Lee, C. C.; Hu, Y.; Ribbe, M. W. *Science* **2010**, *329*, 642.
(4) Hu, Y.; Lee, C. C.; Ribbe, M. W. *Science* **2011**, *333*, 753–755.
(5) Kowalska, J. K.; Hahn, A. W.; Albers, A.; Schiewer, C. E.; Bjornsson, R.; Lima, F. A.; Meyer, F.; DeBeer, S. *Inorg. Chem.* **2016**, *55*, 4485–4497.
(6) Bjornsson, R.; Lima, F. A.; Spatzal, T.; Weyhermüller, T.; Glatzel, P.; Bill, E.; Einsle, O.; Neese, F.; DeBeer, S. *Chem. Sci.* **2014**, *5*, 3096–3103.
(7) Rees, J. A.; Bjornsson, R.; Schlesier, J.; Sippel, D.; Einsle, O.; DeBeer, S. *Angew. Chemie Int. Ed.* **2015**, *54*, 13249–13252.
